# Identification and functional analysis of long non-coding RNAs in human and mouse early embryos based on single-cell transcriptome data

**DOI:** 10.18632/oncotarget.11304

**Published:** 2016-08-16

**Authors:** Jia-jun Qiu, Zhao-rui Ren, Jing-bin Yan

**Affiliations:** ^1^ Shanghai Children's Hospital, Shanghai Institute of Medical Genetics, Shanghai Jiao Tong University School of Medicine, Shanghai 200040, China; ^2^ Key Laboratory of Embryo Molecular Biology, Ministry of Health of China and Shanghai Key Laboratory of Embryo and Reproduction Engineering, Shanghai 200040, China

**Keywords:** long non-coding RNAs (lncRNAs), early embryo, WGCNA, single-cell RNA-seq, human embryogenesis

## Abstract

Epigenetics regulations have an important role in fertilization and proper embryonic development, and several human diseases are associated with epigenetic modification disorders, such as Rett syndrome, Beckwith-Wiedemann syndrome and Angelman syndrome. However, the dynamics and functions of long non-coding RNAs (lncRNAs), one type of epigenetic regulators, in human pre-implantation development have not yet been demonstrated. In this study, a comprehensive analysis of human and mouse early-stage embryonic lncRNAs was performed based on public single-cell RNA sequencing data. Expression profile analysis revealed that lncRNAs are expressed in a developmental stage–specific manner during human early-stage embryonic development, whereas a more temporal-specific expression pattern was identified in mouse embryos. Weighted gene co-expression network analysis suggested that lncRNAs involved in human early-stage embryonic development are associated with several important functions and processes, such as oocyte maturation, zygotic genome activation and mitochondrial functions. We also found that the network of lncRNAs involved in zygotic genome activation was highly preservative between human and mouse embryos, whereas in other stages no strong correlation between human and mouse embryo was observed. This study provides insight into the molecular mechanism underlying lncRNA involvement in human pre-implantation embryonic development.

## INTRODUCTION

Understanding human pre-implantation development can not only provides insight into common human birth defects but also improve our understanding of the pathogenic mechanisms of many complex diseases such as Rett syndrome, Beckwith-Wiedemann syndrome and Angelman syndrome [1, 2]. Thus, it is meaningful to understand the molecular mechanisms underlying pre-implantation development.

Members of numerous non-coding RNA classes are expressed in the oocyte and pre-implantation embryo, and they have have an important role in fertilization and proper embryonic development [3], including directing cell fate decisions and cell differentiation during embryogenesis, which involves the formation of highly complex tissues comprised of many different cell types with specific and stable gene expression patterns [4]. Regulation of non-coding RNA occurs from the beginning of embryonic development. For example, the primary transcript of miR-209~295, which is a miRNA cluster typically associated with the pluripotent state, is first detected in 4- to 8-cell embryos [5].

Long non-coding RNAs (lncRNAs), which are typically over 200 nucleotides in length, are involved in the cleavage stage of embryonic development [6]. Xist, the first identified lncRNA, is sufficient to trigger *cis*-inactivation of the X chromosome during the 4-cell stage, and another lncRNA; Fendrr, mediates long-term epigenetic marks to define expression levels of its target genes in mammalian embryogenesis [6–8]. Notably, a large number of human developmental disorders are related to the abnormal expression of some lncRNAs, such as *DBE-T* in facioscapulohumeral muscular dystrophy, *SNORD115* and *SNORD116* in Prader-Willi Syndrome and *KCNQ1OT1* and *H19* in Beckwith-Wiedemann Syndrome and Silver-Russell Syndrome, which suggests that lncRNAs may play an important role in pre-implantation development [9–11]. However, the expression profiles and the regulation mechanism of lncRNAs in human early-stage embryos remain unclear.

Considering the limited availability of human oocytes and pre-implantation stage embryos, most studies of the functions of lncRNAs involved in embryonic development are based on model animals [12, 13]. A study of zebrafish embryogenesis found that a number of lncRNAs are involved in specific pathways and functions, ranging from cell cycle regulation to morphogenesis [12]. However, substantial differences in gene expression patterns exist between humans and model animals, which may limit the extrapolation of some findings to human embryonic development, especially for lncRNAs for which the sequence conservation is very low [1, 14]. Nevertheless, the differences between lncRNA expression patterns and functions between human and model animals have not yet been clearly elucidated.

In this study, we elucidate the expression profiles and functions of lncRNAs in human early-stage embryos based on single-cell RNA sequencing (RNA-seq) data. We also compare the lncRNA expression profiles of human and mouse early-stage embryos. Genome-wide analysis of the functions of lncRNAs in pre-implantation stage will improve our understandings on the molecular mechanisms of human embryogenesis and developmental disorders.

## RESULTS

### Transcriptome reconstruction from the single-cell RNA-seq data

**Figure 1:**
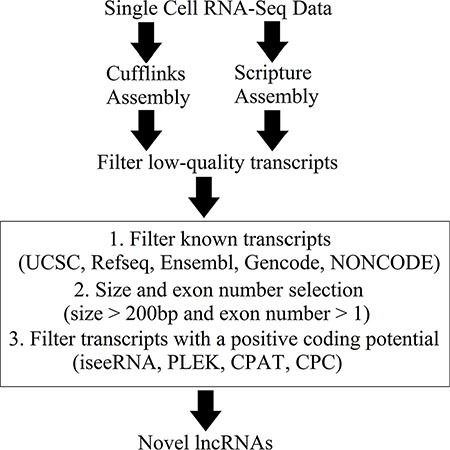
Overview of the novel lncRNA detection pipeline

All reads of the 90 single-cell RNA-seq datasets (GSE36552) were aligned to the human genome (hg19) using HISAT, and the details of the mapping results are shown in Table S1. The mapped reads were assembled into transcripts with the *ab initio* assembly software Cufflinks and Scripture (Figure 1, Table S1). Mouse single-cell RNA-seq data (GSE44183) were aligned to the mouse genome (mm9) (Table S1).

Subsequently, low-quality transcripts were removed using a read coverage threshold (see Materials and Methods). The corresponding receiver operating characteristic (ROC) curves are shown in Figure S1. High-confidence transcripts were retained for downstream analysis.

### Identification of novel lncRNAs

A novel lncRNA detection pipeline was developed to identify novel lncRNAs from the high-confidence transcripts. First, there were 94,418 and 77,464 unannotated transcripts were assembled by Cufflinks and Scripture, respectively. Among them, 535 transcripts which were assembled by both Cufflinks and Scripture were retained for downstream analysis. After size and exon number selection, 452 transcripts were selected as the putative novel lncRNAs. Finally, 421 transcripts were identified as novel lncRNAs based on their low coding potential as calculated with four different prediction tools (Figure 1 and Dataset S1). These novel lncRNAs are listed in Dataset S1.

### Transcriptional profiles across different stages

**Figure 2:**
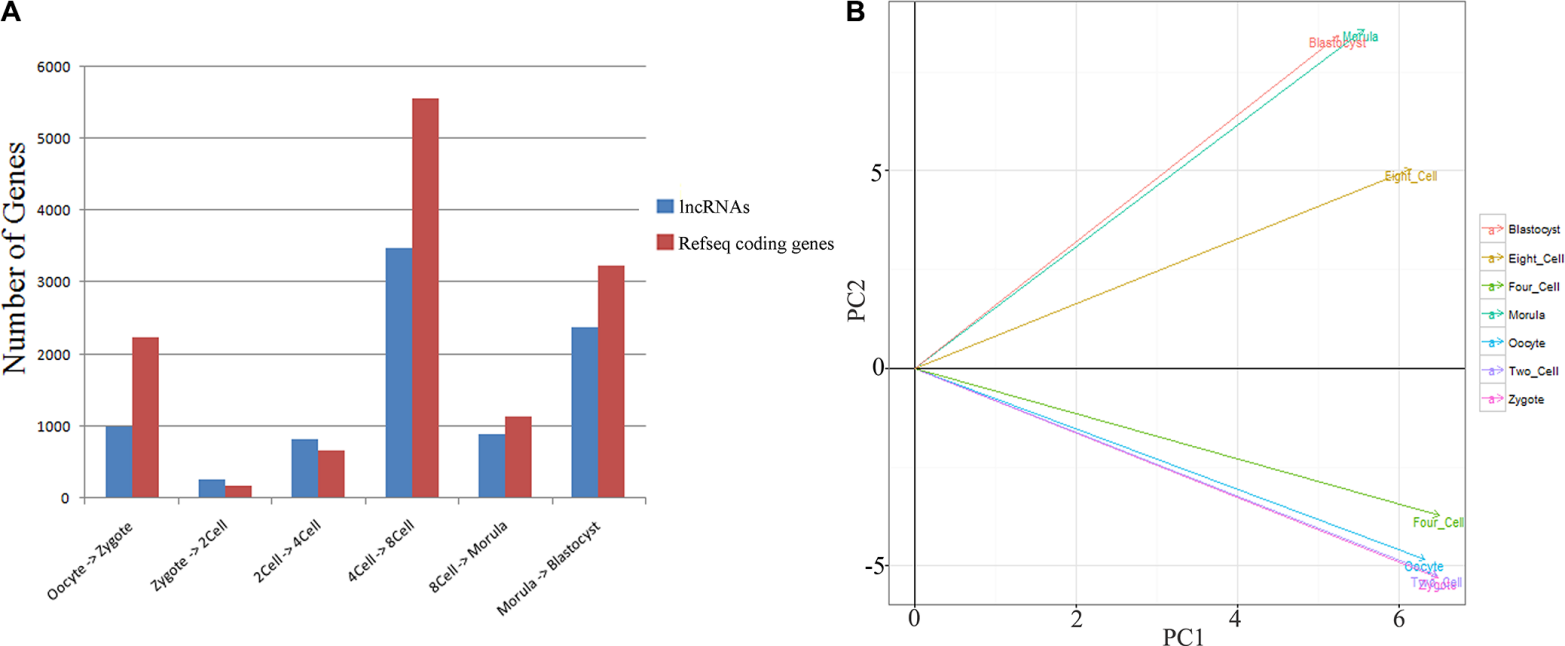
Global gene expression patterns during the seven consecutive stages of human pre-implantation development (**A**) Bar graph showing the total number of differentially expressed genes between successive developmental stages (*q*-value < 0.01 and log_2_ fold change > 1). (**B**) Principal component (PC) analysis based on lncRNA expression profiles of human pre-implantation embryos.

We found that 15,400 and 6063 genes showed stage-specific expression (differential expression between any two consecutive stages) in the human and mouse datasets, respectively. There were notable differences in the human gene expression profiles between the 4- and 8-cell stages (Figure 2), which was consistent with the major maternal-zygotic transition [15, 16]. Accordingly, significant differences were identified between the expression profiles of mouse pronuclei and the 2-cell stage (Figure S2). Two other dramatic changes were also authenticated in human transcript profiles. One was between the oocyte and zygote stages and the other was between the morula to late blastocyst at hatching stages (Figure 2A), which were likely caused by fertilization and cell differentiation, respectively.

### A more temporal-specific expression pattern was identified in lncRNAs rather than in protein-coding genes

**Figure 3:**
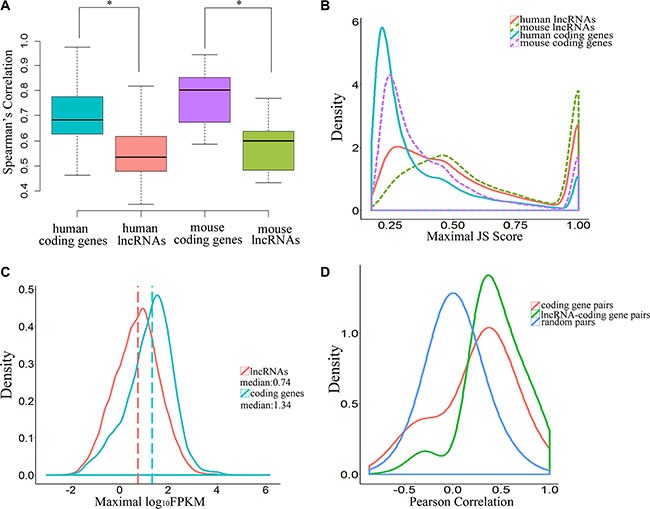
Temporal-specific expression of lncRNAs (**A**) Boxplot indicating the distribution of Spearman's rank correlation coefficients between each embryonic sample pair derived from lncRNAs and coding genes (* means *P*-value < 0.05). (**B**) Distribution of JSD-based specificity of genes in various stages. (**C**) Distribution of maximal expression (log_10_-normalized FPKM counts estimated by Cufflinks) of lncRNAs and coding genes in human pre-implantation development. (**D**) Pearson correlation coefficient distributions for expression levels across the samples in human pre-implantation development. The random pairs are 10,000 random pairs of protein-coding genes.

Previous studies have shown that lncRNAs are expressed in a tissue type–specific manner and that their expression levels are significantly lower than those of protein-coding genes [17]. In this study, the Spearman's rank correlation coefficients derived from lncRNA expression data were significantly lower than those of protein-coding genes, both in human and mouse embryos (*P*-value = 2.2 × 10^−16^, two-tailed Mann-Whitley-Wilcoxon test; Figure 3A, Dataset S2 and Dataset S3), indicating that expression of lncRNAs was more variable than that of protein-coding genes in human early-stage embryonic development. Our analysis showed that the distributions of maximal JS (Jensen-Shannon, temporal specificity) scores for lncRNAs and protein-coding genes were significantly different, and lncRNAs were expressed in a more temporal-specific manner (*P*-value < 2.2 × 10^−16^, Kolmogorov-Smirnov test; Figure 3B, Dataset S4 and Dataset S5) in both human and mouse embryos. Using JS score = 0.5 as a cutoff value, we found that 45.4% of lncRNAs were temporal-specific, relative to only 19.2% of protein-coding genes in humans (*P*-value < 0.001, Fisher exact test). Our data also showed that temporal specificity scores for lncRNAs in mouse were significantly higher than those in human (*P*-value < 2.2 × 10^−16^, Kolmogorov-Smirnov test; Figure 3B, Dataset S4 and Dataset S5). However, the expression levels of lncRNAs were much lower than those of protein-coding genes (*P*-value < 2.2 × 10^−16^, Kolmogorov-Smirnov test; Figure 3C). Together, these observations suggest that lncRNAs exhibit more temporal specificity than protein-coding genes in human early embryos, and these differences were more pronounced in mouse early-stage embryonic development.

### lncRNAs may regulate gene transcription in cis in human embryo development

Some lncRNAs may act in *cis* and affect the expression of genes in their chromosomal neighborhood [18–20]. To test whether lncRNAs act in *cis* in human embryonic development, correlations between the expression patterns of lncRNAs and their neighbor coding genes, including 9440 unidirectional pairs and 3616 bidirectional pairs, were calculated. The results indicated a more correlation between lncRNAs and their coding neighbors than protein-coding gene–protein-coding gene pairs (*P*-value < 2.2 × 10^−16^, Kolmogorov-Smirnov test; *P*-value < 2.2 × 10^−16^, Student's *t*-test, effect size = 0.61; Figure 3D). To confirm that this was a true *cis* effect of lncRNAs, we analyzed the correlations between the expression patterns of lncRNAs and their protein-coding gene neighbors and between protein-coding gene neighbors in two situations. The correlation between lncRNAs and protein-coding gene neighbors was significantly higher than between protein-coding genes and their protein-coding gene neighbors for both unidirectional neighbor-gene pairs (*P*-value < 2.2 × 10^−16^, Kolmogorov-Smirnov test; *P*-value < 2.2 × 10^−16^, Student's *t*-test, effect size = 0.64; mean correlation: 0.423 for lncRNA–protein-coding gene pairs vs. mean correlation: 0.233 for protein-coding gene–protein-coding gene pairs; Figure S3A) and divergent neighbor gene pairs (*P*-value < 2.2 × 10^−16^, Kolmogorov-Smirnov test; *P*-value < 2.2 × 10^−16^, Student's *t*-test, effect size = 0.61; mean correlation: 0.460 for lncRNA–protein-coding gene pairs vs. mean correlation: 0.251 for protein-coding gene–protein-coding gene pairs; Figure S3B).

Taken together, these results confirmed that there were remarkably different expression patterns between lncRNA–protein-coding gene pairs and neighboring protein-coding gene pairs in both directions, which revealed that lncRNAs may regulate gene transcription in *cis* in human early-stage embryos.

### Functions of lncRNAs in human early-stage embryonic development

**Figure 4:**
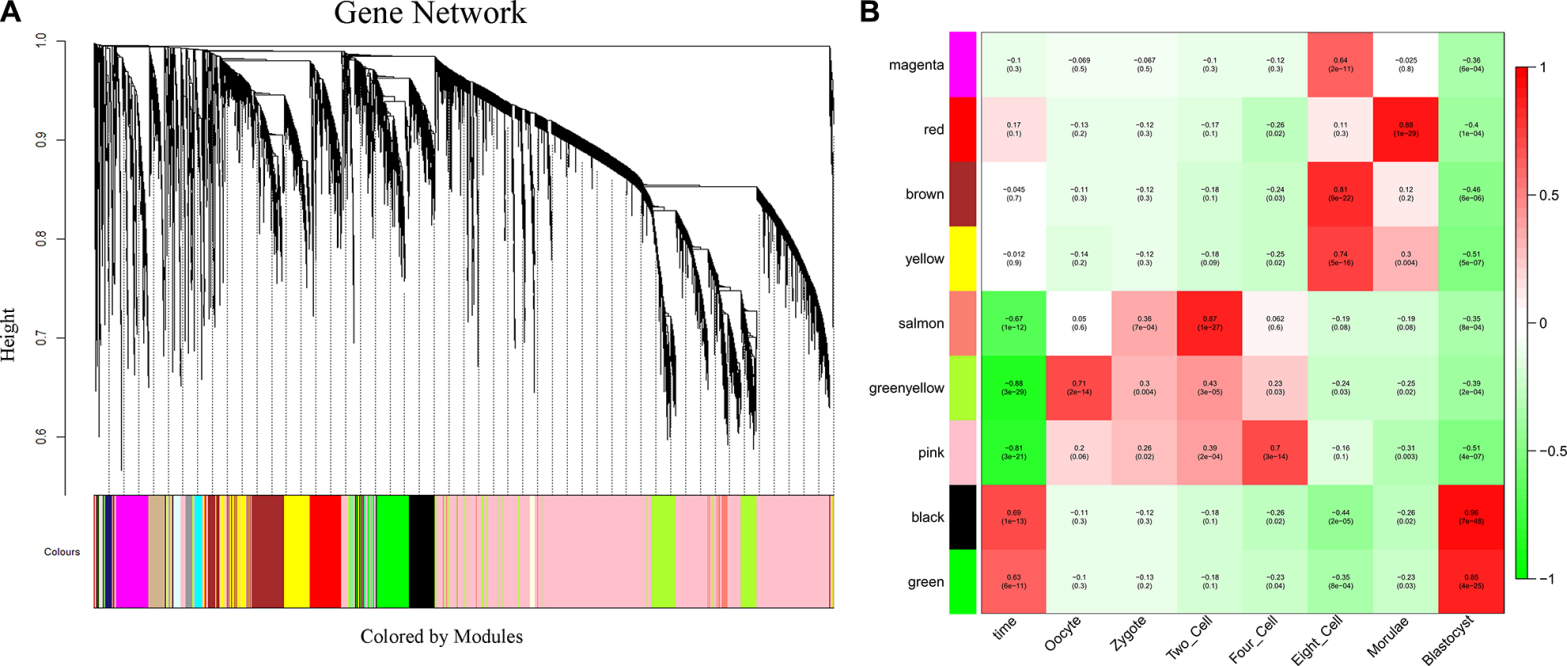
Network analysis of human pre-implantation development (**A**) Hierarchical cluster tree showing co-expression modules identified using WGCNA. Modules correspond to branches and are labeled by colors as indicated by the color band underneath the tree. (**B**) Heatmap of correlations followed by the *P*-values in parentheses between modules and developmental stage. The color of each square corresponds to the degree of correlation: positive correlation, red; negative correlation, green; no correlation, white. The “time” column on the left represents the correlation of each module with the entire development process.

**Figure 5:**
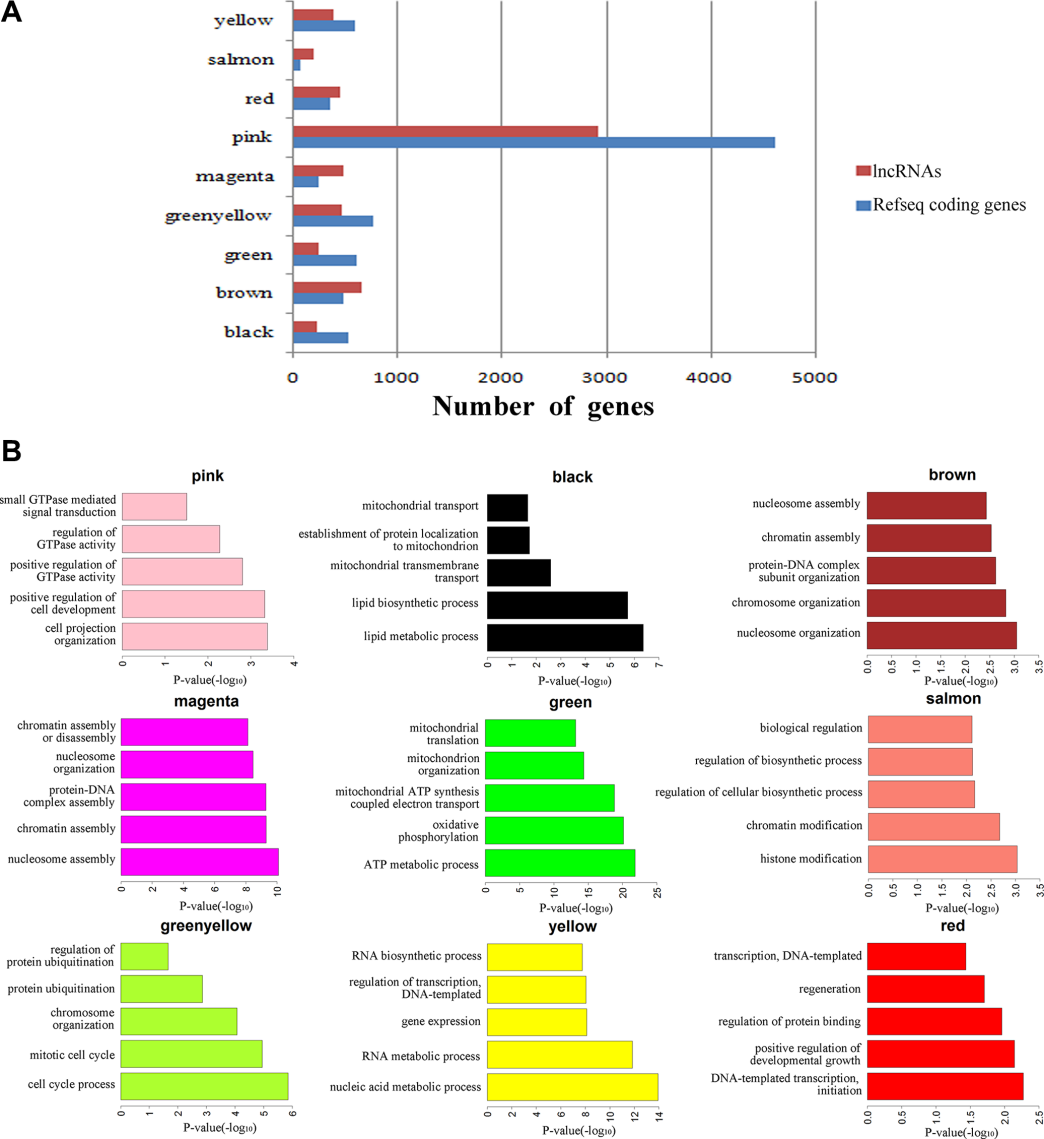
Function prediction of lncRNAs involved in pre-implantation development (**A**) Bar graph showing the number of lncRNAs and coding genes in each module. (**B**) Bar plots showing GO enrichment in the modules. The length of the bars indicates the significance.

**Figure 6:**
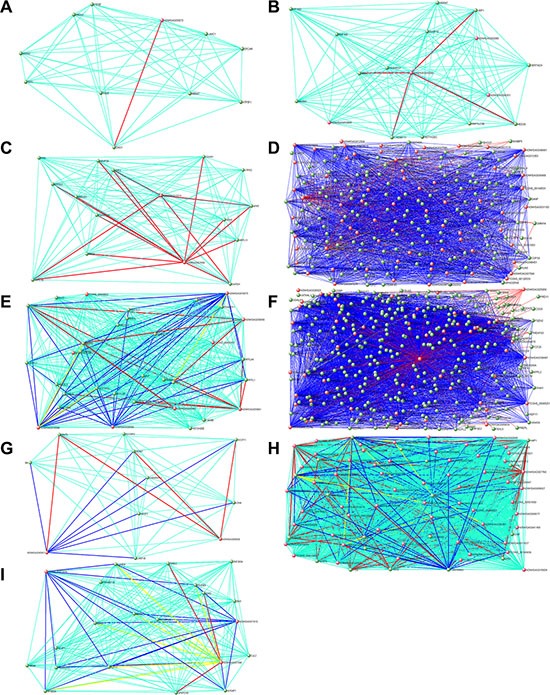
Hub gene networks for human stage-specific modules Visualization of gene-gene interactions within each module. The connections were drawn using the VisANT tool. The genes with at least one connection when the weighted cutoff value was ≥ 0.1 are shown. Each node represents a hub gene. The red nodes are hub lncRNAs. The green edges mean co-expression, and the red edges mean both co-expression and interaction, and the blue edges mean both co-expression and neighbor gene, and the yellow edges mean all three situation (co-expression, interaction and neighbor gene). To make the background clear, the green edges are not shown in Figure D and F. (**A**) Black module. (**B**) Brown module. (**C**) Green module. (**D**) Greenyellow module. (**E**) Magenta module. (**F**) Pink module. (**G**) Red module. (**H**) Salmon module. (**I**) Yellow module.

To investigate the potential roles of lncRNAs in early-stage embryonic development, weighted gene co-expression network analysis (WGCNA) was performed on the stage-specific genes (Dataset S6 and Dataset S7). This analysis identified 17 modules in human embryos, 9 of which were highly correlated (correlation > 0.6, *P*-value < 10^−4^) with specific developmental stages or with the entire developmental process (Figure 4, Figure S4 and Dataset S8). In mouse embryos, six of eight modules were highly correlated with early embryonic development (Figure S5, Figure S6 and Dataset S9). All of the modules contained a large number of lncRNAs (Figure 5A, Dataset S8 and Dataset S9). Enrichment analysis of GO terms and KEGG pathways within the modules was conducted (Figure 5B, Dataset S10 and Dataset S11). We also found that most of coding genes were the neighbor genes of lncRNAs in every modules (*P*-value = 0.004, Table 1), which validated the former result that lncRNAs may regulate gene transcription in *cis* in human early-stage embryos. Besides, we performed a hub-gene network analysis of each stage-specific module, and the interaction between hub lncRNAs and hub coding genes was also analyzed. The hub-lncRNAs were found in all stage-specific modules (Figure 6, Dataset S8 and Dataset S9), while some hub lncRNAs were found to co-localize with hub coding genes and *cis*-regulate them, and the others were confirmed to bind directly with the hub coding genes and *trans*-regulate them in almost all stage related modules (Figure 6, Dataset S12 and Dataset S13). The functions of these lncRNAs can be predicted based on the hub genes of known biological functions with which they were co-expressed with or bound.

**Table 1:** Coding genes were the neighbor genes of lncRNAs in every modules

Module	Neighbor coding genes (%)	Non- neighbor coding genes (%)	*t*	*P*-value
black	56.23	43.77	3.970	0.004
brown	59.46	40.54		
green	54.13	45.87		
greenyellow	55.20	44.80		
magenta	52.81	47.19		
pink	63.21	36.79		
red	57.43	42.57		
salmon	47.37	52.63		
yellow	56.57	43.43		
Mean	55.82 ± 4.4	44.18 ± 4.4		

### lncRNAs regulate human oocyte maturation

One human module that contained a large number of lncRNAs (greenyellow; 464 lncRNAs and 759 protein-coding genes; Figure 5A) was highly correlated with the oocyte stage (Figure 4B). Genes in this module were enriched in the oocyte meiosis pathway (KEGG, *P*-value = 0.005, Dataset S10). There were many hub coding genes which were related to oocyte maturation co-expressed with hub lncRNAs. Some of them were bound directly by hub lncRNAs, such as *AURKA*, *BCL2L10,* and the others such as *TNFSF13,* were the neighbor genes of hub lncRNAs [21–23] (Figure 6, Dataset S8, Dataset S12). Thus, hub lncRNAs of this module may be important for the regulation of oocyte maturation. Notably, lncRNAs in this module are related to protein ubiquitination (GO enrichment, *P*-value = 0.001; Figure 5B and Dataset S10) and regulation of protein ubiquitination (GO enrichment, *P*-value = 0.02; Figure 5B and Dataset S10), which agrees with previous reports that the ubiquitin-proteasome pathway (UPP) can control oocyte meiotic maturation [24, 25]. These results suggested that lncRNAs may activate oocyte maturation and meiosis.

### lncRNAs involved in human zygotic genome activation

Zygotic genome activation (ZGA) occurs between the 4- and 8-cell stages of human embryonic development and is the point at which zygotic transcripts gradually take control of development as maternal transcripts are degraded [1, 26]. In our study, half of human stage-specific modules were related to the 4- and 8-cell stages, and these modules contained a large fraction of lncRNAs. Genes in the human modules highly correlated with the 4-cell stage (pink) were enriched in GTPase, which mediates signal transduction (Figure 5B and Dataset S10). There were many hub coding genes which were related to GTPase co-expressed with hub lncRNAs. Some of them were bound directly by hub lncRNAs, such as *RASA3*, and the others such as *GNG2,* were the neighbor genes of hub lncRNAs (Figure 6, Dataset S8, Dataset S12). The human modules related to the 8-cell stage (magenta, brown and yellow) were enriched in several functions related to ZGA, including nucleosome assembly (GO enrichment, *P*-value = 8.19 × 10^−11^; Figure 5B and Dataset S10) and chromatin assembly (GO enrichment, *P*-value = 5.05 × 10^−10^; Figure 5B and Dataset S10). In conclusion, we found that some hub lncRNAs may activate ZGA during the human 4- and 8-cell stages.

### lncRNAs regulate mitochondrial functions

Basic research in model species and clinical *in vitro* fertilization studies have shown that mitochondria play an important role in the regulation of mammalian early embryogenesis and that embryonic mitochondrial replication occurs after the hatched-blastocyst stage [27, 28]. In this study, we found that the gene functions in two human blastocyst stage–related modules (black and green) were both enriched in mitochondrion functions (Figure 5B and Dataset S10). Hub lncRNAs in these two modules bound directly and co-expressed with several mitochondrial function genes, including *ATP5G3*, *COX4I1* and *NDUFS6*, which indicated that hub lncRNAs of blastocyst modules may correlate to mitochondrial function (Figure 6, Dataset S8, Dataset S12) [29].

### Comparison of lncRNA functions between human and mouse early-stage embryonic development

**Figure 7:**
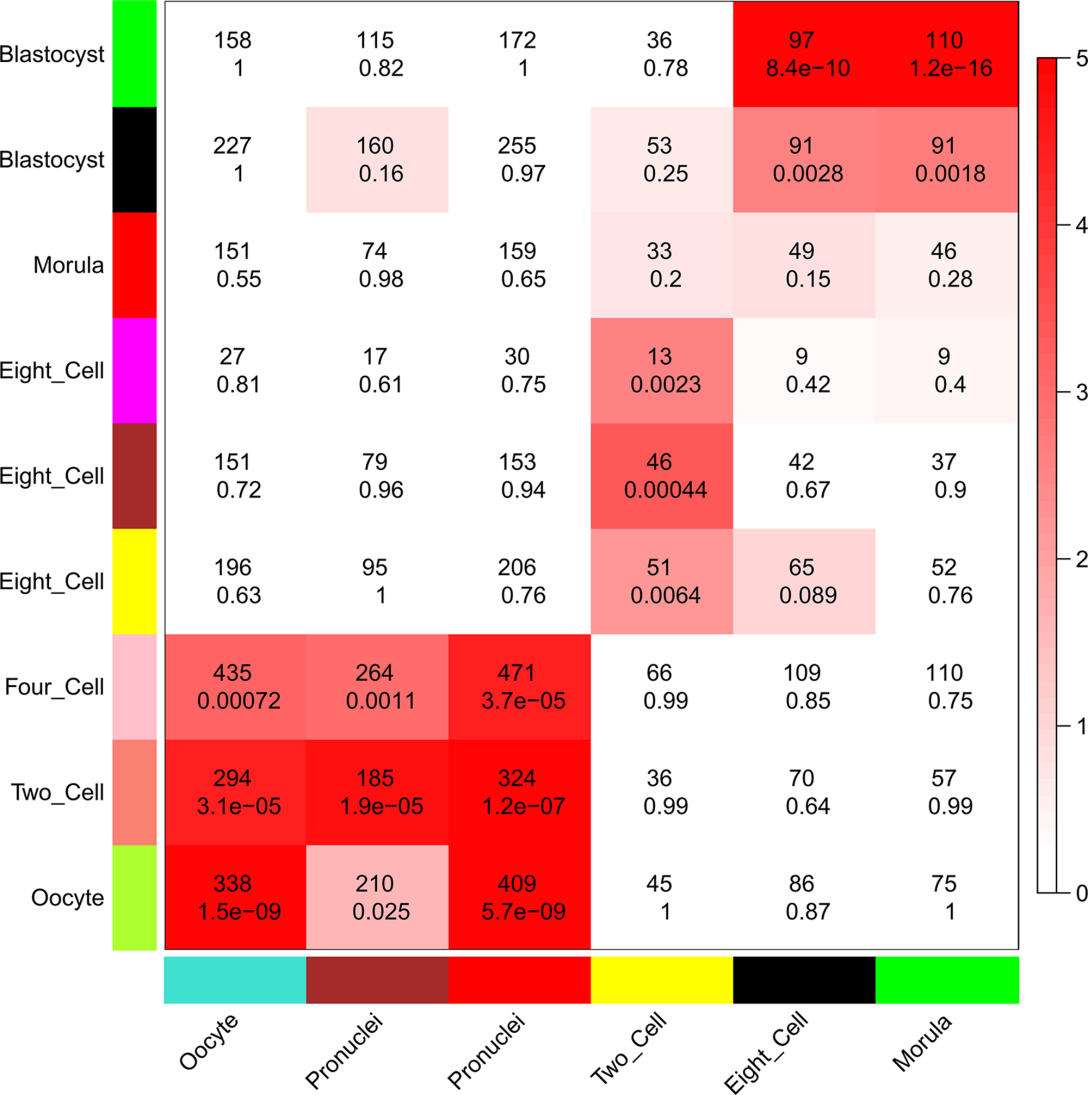
Comparison of modules in human and mouse early embryonic development Heatmap showing the significance of gene overlaps between independently constructed human and mouse modules. The *x* axis shows only mouse stage-specific modules (*n* = 6), and the *y* axis shows all human modules (*n* = 9). Each cell contains the number of intersecting genes and the *P*-value of the intersection. Color legend represents −log_10_–transformed *P*-values based on a hypergeometric test.

Because we found that lncRNAs were expressed in a more temporal-specific manner in mouse early embryos than that in human embryos, we also analyzed the functions of lncRNAs in mouse early embryonic development. Though lncRNAs were also found to be related to several functions in specific stages during mouse early embryonic development, the functions of lncRNAs were not identical in mouse or human embryonic development. For example, lncRNAs in the human 8-cell stage appear to have an important role in GTPase mediated signal transduction (Figure 5B and Dataset S10), whereas lncRNAs in the mouse 8-cell stage do not appear to have such functions (Dataset S11). Comparing the human modules to the mouse developmental data, we found that the human 8-cell modules overlapped significantly with the mouse 2-cell module, during which mouse ZGA occurs (Figure 7). While mouse pre-major ZGA genes are spread over the longer gestational pre-major ZGA stage in humans. Likewise, post-major ZGA networks are found to have significant overlap and spread throughout all post-major ZGA human stages (Figure 7). These results suggested that the networks (modules) of lncRNAs involved in ZGA are particularly conserved between human and mouse embryos, but there is less preservation across other stages.

## DISCUSSION

A number of diseases are caused by the disruption of epigenetic regulation during early embryonic development [3, 30]. However, no systematic studies focused on the functions of lncRNAs during human early embryonic development has been described. Here we identified 421 novel lncRNAs in the first study to determine expression levels of all genes in human early-stage embryos. Furthermore, we found that lncRNAs are expressed in a developmental stage–specific manner, and they may regulate gene transcription in *cis* in human embryonic development.

Oocyte meiosis is a specialized cell cycle that gives rise to fertilizable haploid gametes and is precisely controlled on many levels. Previous studies have found that the UPP regulates both human and mouse oocyte meiotic maturation in several ways [25, 31]. CRL4-DCAF1 ubiquitin E3 ligase facilitates oocyte meiotic maturation by proteasomal degradation of the protein phosphatase 2A scaffold subunit PP2A-A, which inhibits cohesin removal and homologous chromosome separation during meiosis I [24]. UPP also has roles in oocyte meiotic maturation, because the degradation of cyclin B1 mediated by UPP is necessary for disjunction of pairs of homologous chromosomes during the first meiotic division in oocytes [25]. In hub-gene network analysis, we found 60 lncRNAs in the regulation network module built on co-expression with 10 UPP genes, among them 30 lncRNAs bound directly with the 9 UPP genes (Figure 6F). Thus lncRNAs may activate oocyte meiotic maturation through regulating the UPP in both human and mouse. With these results, we have a better understanding of the mechanisms of oocyte meiotic maturation and oocyte maturation failure [32].

The ZGA is another important early embryonic developmental period in which maternal mRNAs are cleared and embryonic transcription is activated [26]. Remodeling of chromatin surrounding nucleosomes, including repositioning of nucleosomes and post-translational modifications of histones, coincides with ZGA, which leads to exposure of the transcription start sites of zygotic genes and permits recruitment of the RNA polymerase II complex [26]. Previous studies have shown that lncRNAs can regulate chromatin remodeling and recruitment of the RNA polymerase II [33, 34]. For example, lncRNA SChLAP1 antagonizes SNF5 (also known as SMARCB1), an essential subunit that facilitates SWI/SNF binding to histone protein [35]. In this study, we confirmed that lncRNAs may stimulate ZGA through regulating nucleosome assembly and chromatin assembly during ZGA.

As the mouse is a wildly used animal model for human disease research, we compared human and mouse stage-specific modules in this study. We found that the networks of lncRNAs in the human 8-cell stage were particularly similar to those in the mouse 2-cell stage, and the pre- and post-ZGA modules in human and mouse overlapped across multiple stages. This probably reflects species-specific differences in human and mouse gestational periods and/or the very low sequence conservation of lncRNAs, because major differences in transcript structure result in functional differences [14]. Because of the large differences between human and mouse lncRNA networks, except those involved in ZGA, the value of research in mice may be limited, and it will be important to examine the functions of lncRNAs in human early embryonic development directly.

## MATERIALS AND METHODS

### Single-cell RNA-seq dataset

The single-cell RNA-seq dataset of human early-stage embryos (GSE36552) was downloaded from the Gene Expression Omnibus (GEO) of the National Center for Biotechnology Information. The dataset consists of 90 samples from seven crucial stages of embryo development: 3 metaphase II oocyte samples, 3 zygote samples, 6 2-cell stage samples, 12 4-cell stage samples, 20 8-cell stage samples, 16 morula stage samples and 30 late blastocyst at hatching stage samples [16]. The single-cell RNA-seq dataset of mouse early-stage embryos (GSE44183), which includes 17 samples ranging from the oocyte to morula stages, was also downloaded from the GEO. Both of these datasets were generated with the Illumina HiSeq 2000 system [36].

### Data pre-processing and filtering

#### Read mapping and transcript assembly

Reads were aligned to the human (hg19) and mouse (mm9) genomes by HISAT (version 1.4.1; a successor to TopHat2), which is the fastest aligning algorithm currently available and one of the most accurate [37].

Aligned reads from HISAT were then assembled into transcripts separately by two different approaches: Cufflinks (version V2.2.1) and Scripture (beta version 2). Cufflinks uses a probabilistic model to simultaneously assemble and quantify the expression level of a minimal set of isoforms and provides a maximum likelihood explanation of the expression data in a given locus. Scripture uses a statistical segmentation model to distinguish expressed loci from experimental noise and uses spliced reads to assemble expressed segments. It reports all statistically significantly expressed isoforms in a given locus. The two approaches might generate different results in terms of assembled transcripts and numbers of products [38].

Cufflinks version V1.0.3 was run with default parameters (and ‘min-frags-per-transfrag = 0’) and Scripture version 1.0 was run with default parameters [38–40].

### Filtering low-quality transcripts

To remove low-quality reconstructed transcripts, transcripts assembled by Cufflinks with coverage below 4.03418 reads per base were eliminated (the threshold of transcripts assembled by Scripture was 1.39501). This minimal read coverage threshold was calculated by the method described previously [17, 41]. Transcripts that recovered 75% of annotation were regarded as good reconstructed transcripts. The ROC curve was used to evaluate the performance of different coverage thresholds between good and bad reconstructed transcripts. The final threshold was the average of the optimum threshold for coding (‘NM’ prefix) and non-coding (‘NR’ prefix) RNAs in RefSeq (NCBI Reference Sequence Database).

### Calculating optimum coverage threshold

A coverage threshold set *T* with specified sensitivity and specificity was generated with the R package pROC based on the coverage values of good and bad reconstructed transcripts [42]. The index of the optimum coverage threshold in set *T* can be obtained by formula 1, in which i* represents the index of the optimum coverage threshold and *sensitivities[i]* and *specificities[i]* respectively denote the sensitivities and specificities of the *i*th coverage threshold. The value for *i* is enumerated in *I,* ranging from 1 to the size of the coverage threshold set *T.* Then the optimum coverage threshold can be obtained with formula 2 [43].

$$i^{*}={\mathrm{argmin}}_{i\in I}\left\{\sqrt{{\left(1-\text{sensitivities}\left[i\right]\right)}^{2}+{\left(1-\text{specificities}\left[i\right]\right)}^{2}}\right\}$$ (1)

$$t^{*}=T\left[i\right]$$ (2)

### Novel lncRNA detection pipeline

The novel lncRNAs were obtained by the following steps: (1) Cuffcompare in Cufflinks was run using default parameters (and ‘-M discard (ignore) single-exon transfrags and reference transcripts’) to combine our transcripts with annotations from five well-established databases, Refseq (ref_GRCh37.p13_top_level.gtf), Ensembl (Ensembl_Homo_sapiens.GRCh37.75.gtf), UCSC (hg19), Gencode (gencode.v19.annotation.gtf) and the lncRNA database NONCODE 4.0 (NONCODEv4u1_human_lncRNA.gtf) [44–49]; (2) unannotated transcripts were acquired based on the overlap of the combined transcripts, and BEDTools (version 2.18) was used to eliminate transcripts that had at least one exon overlapping with annotations from any of the five databases; (3) transcripts > 200 bp were then selected [16, 50] and (4) novel lncRNAs were acquired based on non-coding potential by integrating the results from the four prediction tools: iseeRNA, CPAT, CPC and PLEK [51–54].

### Estimating relative expression and differential expression analysis

A matrix of gene expression levels across all samples was obtained by computing the expression levels of Refseq coding genes and lncRNAs (both novel lncRNAs and the annotated lncRNAs) with Cuffquant and Cuffnorm [39]. The annotations of Refseq coding genes and annotated lncRNAs were directly downloaded from the highly reliable database: Refseq and NONCODE V4.

The R package Monocle was used to conduct differential expression tests between any two consecutive stages. Differential expression of a specific gene between any two consecutive stages was noted if the log_2_ fold change was >1 and the false discovery rate–adjusted *P*-value indicated statistical significance (*q*-value < 0.01) after the Benjamini-Hochberg correction for multiple testing [43, 55].

### Temporal specificity analysis

#### Normalization of expression vectors for temporal specificity calculation

To calculate the temporal specificity scores of genes, the expression vector needed to be converted to an abundance density. First, the raw FPKM (fragments per kilobase of exons per million fragments mapped) of each gene was converted to log_10_(FPKM+1). Then this expression vector was normalized to a density vector by formula 3, in which *V* = (v_1_,…,v_n_) is the original raw FPKM abundance estimation of each gene and *V*' is the new normalized density vector [41].

$$V'=\frac{\log_{10}\left(V+1\right)}{\sum_{i=1}^{n}\log_{10}\left(v_{i}+1\right)}$$ (3)

#### Calculating temporal specificity score

To calculate the temporal specificity score, we used an entropy-based measure to quantify the similarity between a gene's expression pattern and another predefined pattern that represents an extreme case in which a gene was expressed during only one stage [41]. The entropy of a discrete probability distribution was calculated by formula 4.

$$\begin{array}{cc} H\left(p\right)=-\sum_{i=1}^{n}p_{i}\log\left(p_{i}\right),s,t & p=\left(p_{1},p_{2},..,p_{n}\right),0\leq p_{i}\leq 1,\sum_{i=1}^{n}p_{i}=1 \end{array}$$ (4)

The temporal specificity score was defined by formula 5, in which *JS*_dist_ dist was the Jensen-Shannon distance (JSD) between two stage expression patterns and e was the gene expression pattern across n stages (formula 6). And *e^s^* was a predefined expression pattern that represented the extreme case in which a gene was expressed in only one stage. It was defined by formula (7).

$$JS_{\text{sp}}\left(e|s\right)=1-JS_{\text{dist}}\left(e,e^{s}\right)$$ (5)

$$JS_{\text{dist}}\left(e^{1}+e^{2}\right)=\sqrt{H\left(\frac{e^{1}+e^{2}}{2}\right)-\frac{H\left(e^{1}\right)+H\left(e^{2}\right)}{2}}$$ (6)

$$\begin{array}{cc} e^{s}=\left(e_{1}^{s},..,e_{n}^{s}\right),s.t & e_{i}^{s}=\left\{\begin{array}{ll} 1 & \text{if }i=s\\ 0 & \text{otherwise} \end{array}\right\} \end{array}$$ (7)

Finally, the temporal specificity score of a gene was defined as the maximal temporal specificity score across all *n* stage of the genes expression pattern *e*:

$$\begin{array}{cc} JS_{\text{sp}}\left(e\right)={\mathrm{argmax}}_{s}JS_{\text{sp}}\left(e|s\right), & s=1..n. \end{array}$$ (8)

#### Neighboring gene correlation analysis

Two genes were defined as neighbors if the distance between the gene bodies was < 10 kb. Correlation between the expression pattern of an lncRNA and its neighbor coding gene was estimated by calculating the Pearson correlation coefficient (*P*-value ≤ 0.05) between their density-normalized expression vectors [17, 41]. The neighbor genes could be divided into two categories: divergent (bidirectional) neighbor gene pairs and unidirectional neighbor gene pairs. The divergent neighbor gene pairs were identified as gene pairs that were arranged head-to-head on opposite strands [56].

### Analysis of lncRNA functions in human early-stage embryonic development

#### lncRNA co-expression network construction and gene module detection

The R package WGCNA was used to construct an lncRNA co-expression network [57]. The stage-specific genes, those that were differentially expressed between any two consecutive stages, were selected to construct the network. A signed weighted correlation network was constructed by first creating a matrix of Pearson correlation coefficients between all pairs of genes across the measured samples. Second, an adjacency matrix was calculated by raising the correlation matrix to power β = 5. The power of 5 was the soft threshold of the correlation matrix and made the adjacency network exhibit approximate scale-free topology (*R*^2^ = 0.9). To minimize effects of noise and spurious associations, the adjacency matrix was transformed into a Topological Overlap Matrix (TOM). Genes with highly similar co-expression relationships were grouped together by performing average linkage hierarchical clustering on the topological overlap. Dynamic Hybrid Tree Cut algorithm was used to cut the hierarchal clustering tree and define modules as branches from the tree cutting. The expression profile of each module was represented by its first principal component (module eigengene), which could explain the most variation in the module expression levels. Modules with highly correlated module eigengenes (correlation > 0.85) were merged together.

#### Identification and visualization of hub genes

The module membership (also known as module eigengene based connectivity, kME) of each gene was calculated by correlating the gene expression profile with module eigengenes with formula 9, in which *x_i_* is the gene expression profile of gene i and *ME^q^* is the module eigengene of the module *q* [57].

$${\text{kME}}_{q}\left(i\right)=\text{cor}\left(x_{i},\;ME^{q}\right)$$ (9)

Genes with the highest module membership values were referred to as intramodular hub genes (kME ≥ 0.9, *P*-value < 10^−22^). Intramodular hub genes, which were centrally located inside the module, represent the expression profiles of the entire module and reflect the core functions of the module [57]. We used VisANT to visualize the gene connections (based on topological overlap) among the intramodular hub genes [58].

#### Interaction analysis of hub genes

LncTar, a reliable bioinformatics tool, was used to analyze the interaction between hub lncRNAs and hub coding genes in each module [59]. The variation on the standard “sliding” algorithm approach was utilized to calculate the normalized binding free energy (ndG) and identify the minimum free energy joint structure. The ndG was regard as a cutoff (ndG ≤ −0.1) to determine the paired RNAs as either interacting or not. The accuracy of LncTar is over 80% confirmed by the biological experiments [59].

### Module preservation statistics

To compare human and mouse lncRNA modules, we mapped human genes to the orthologous mouse gene annotations from the Mouse Genome Informatics (MGI) database [60]. The function ‘overlapTableUsingKME’ in the WGCNA R package was used to assess whether two modules were preserved based on a hypergeometric test that uses kME [61].

### Function enrichment analysis

Gene Ontology (GO) enrichment and Kyoto Encyclopedia of Genes and Genomes (KEGG) pathway analysis of modules was carried out with the R packages GOstats and org.Hs.eg.db. Hypergeometric tests were applied with a *P*-value cut-off of 0.05 and minimum gene count of 5. Each module was tested for GO enrichment in terms of the Biological Process categories [62, 63].

## SUPPLEMENTARY MATERIALS FIGURES AND TABLES






























