# Supplementary material for: Identification and functional analysis of long non-coding RNAs in human and mouse early embryos based on single-cell transcriptome data
Source: Oncotarget. 2016 Aug 16;7(38):61215–28. doi: 10.18632/oncotarget.11304 (PMC5308646; doi:10.18632/oncotarget.11304)
Supplement: Supplementary file 1 [file oncotarget-07-61215-s001.pdf]

Identification and functional analysis of long non-coding RNAs in human and mouse early embryos based on single-cell transcriptome data

Supplementary Materials

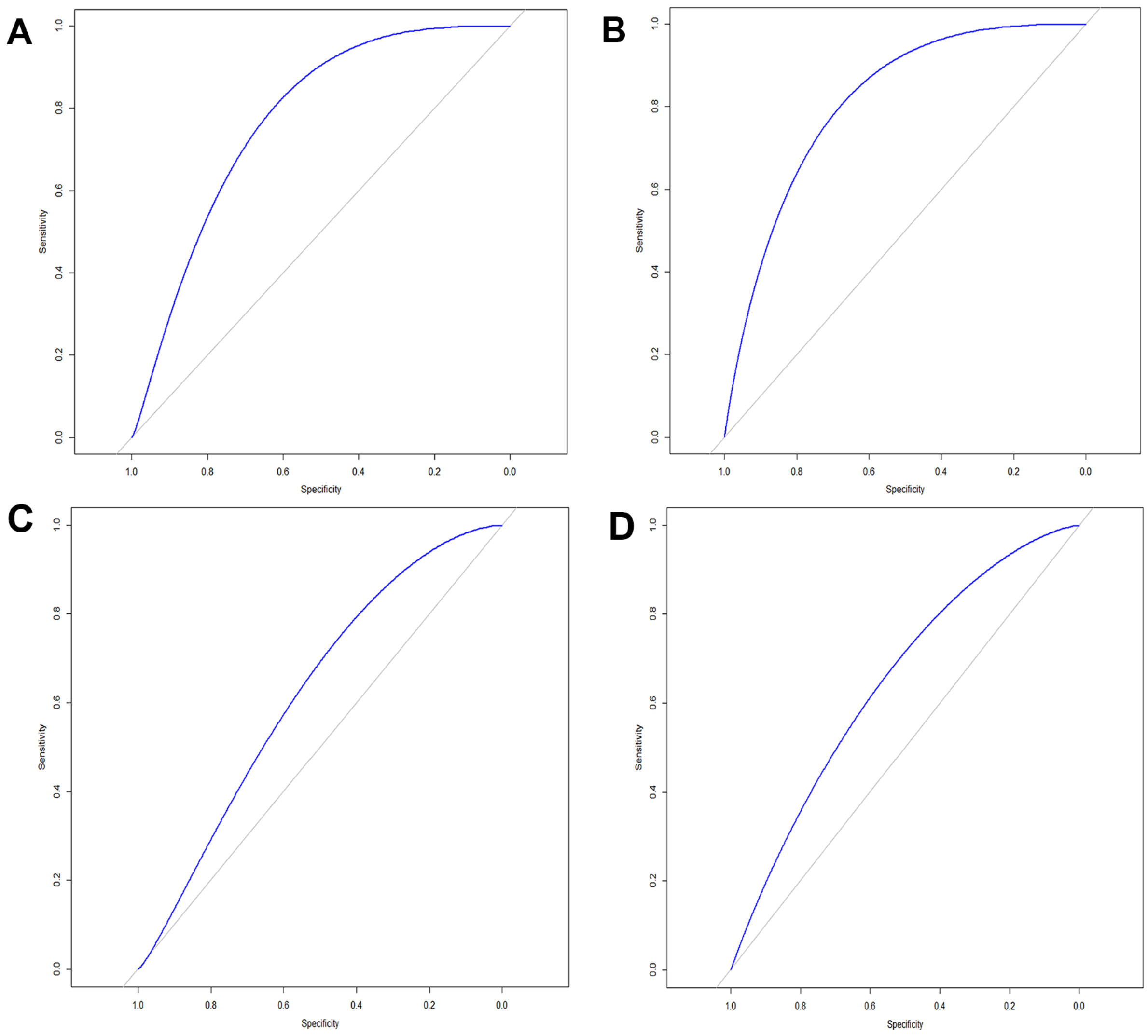

Supplementary Figure S1: Performance of coverage threshold in distinguishing between high- and low-quality transcripts. (**A**) mRNA transcripts assembled by Cufflinks (**B**) lncRNA transcripts assembled by Cufflinks. (**C**) mRNA transcripts assembled by Scripture (**D**) lncRNA transcripts assembled by Scripture. The blue ROC curve represents the performance of coverage thresholds in classifying the complete and partial transcripts. The corresponding area under the curve values were 0.7711 and 0.8115 for mRNA and lncRNA assembled by Cufflinks , respectively. And the corresponding area under the curve values were 0.6233 and 0.6482 for mRNA and lncRNA assembled by Scripture, respectively.

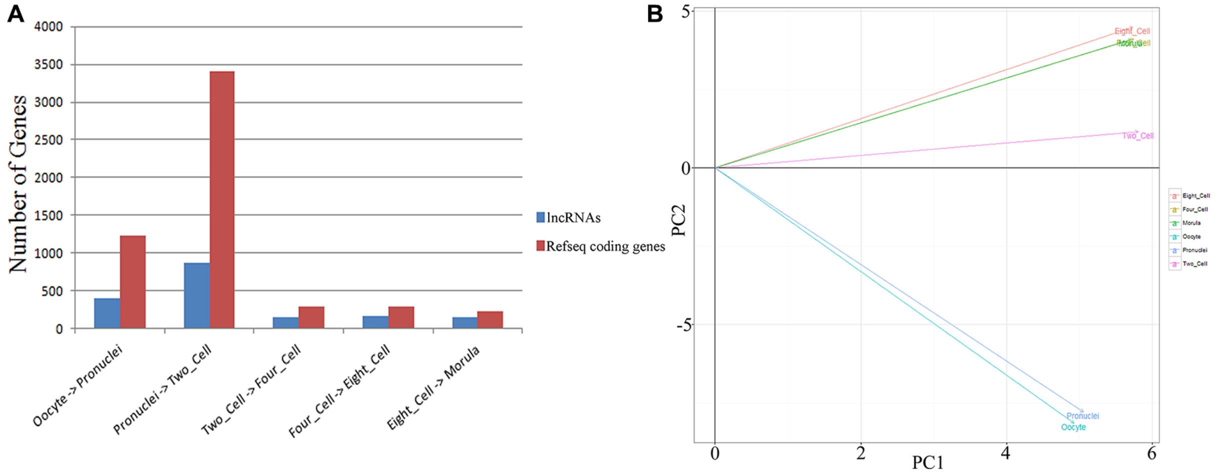

Supplementary Figure S2: Global gene expression patterns during six consecutive stages of mouse pre-implantation development. (**A**) Bar graph showing the total number of differentially expressed genes between successive developmental stages (*q*-value < 0.01 and log2 fold change > 1). (**B**) Principal component (PC) analysis based on lncRNA expression profiles of mouse pre-implantation embryos.

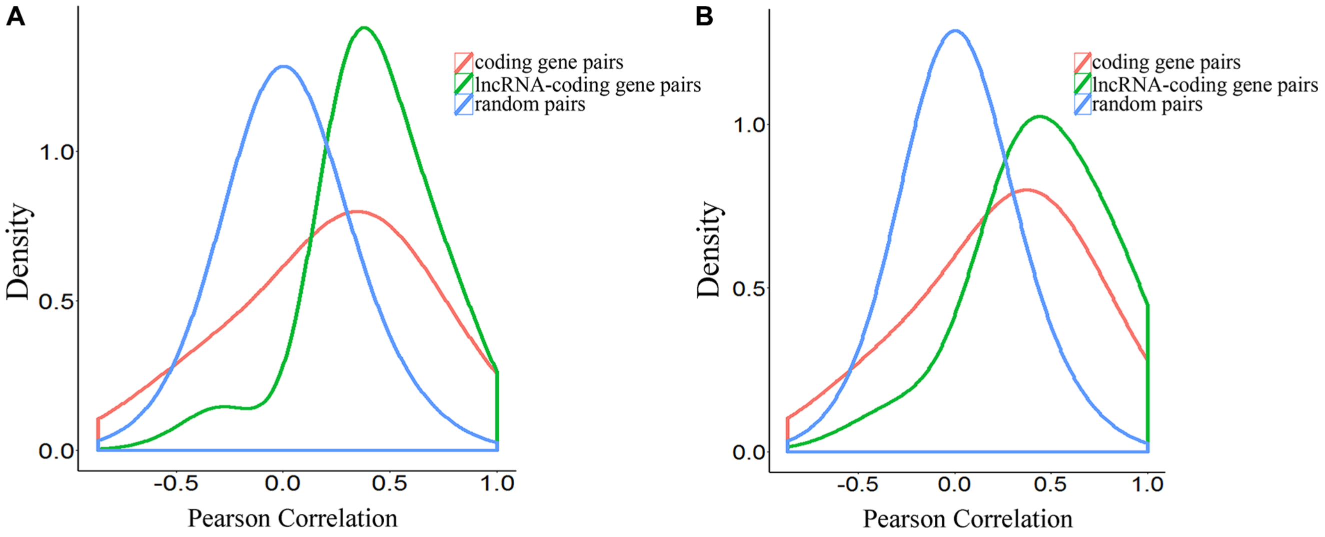

Supplementary Figure S3: Correlation of expression patterns between pairs of neighboring genes. Distributions of Pearson correlation coefficients for expression levels across samples for (**A**) unidirectional and (**B**) bidirectional gene pairs in human pre-implantation development. The random pairs are 10,000 random pairs of protein-coding genes.

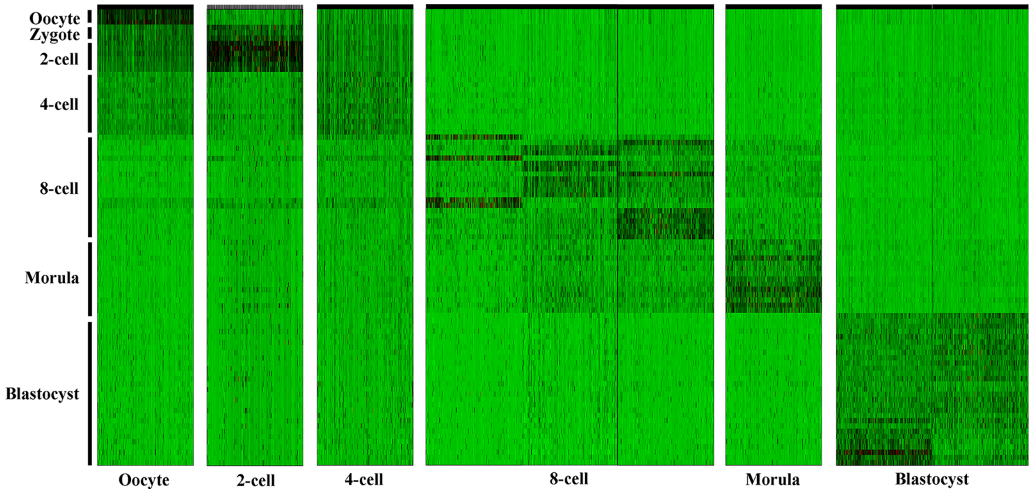

Supplementary Figure S4: Expression of human stage-specific genes. Heatmap showing expression pattern of human stage-specific genes in 9 representative stage-specific modules across all samples. The *x* axis shows the stage to which the module is highly correlated, and the *y* axis shows the whole developmental process.

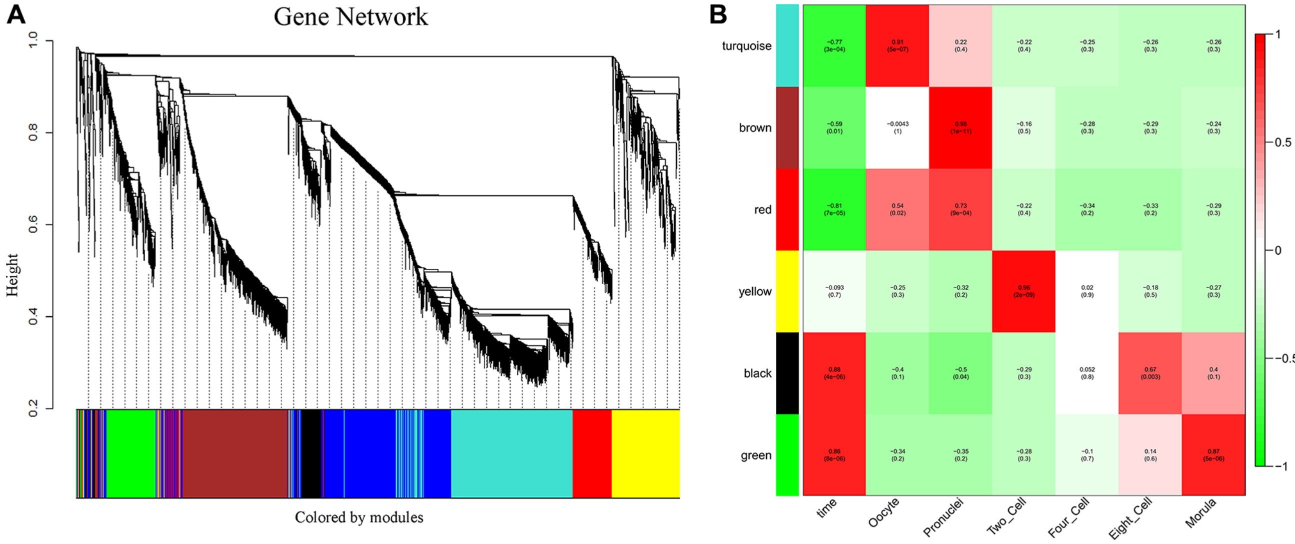

Supplementary Figure S5: Network analysis of mouse pre-implantation development. (**A**) Hierarchical cluster tree showing co-expression modules identified using WGCNA. Modules correspond to branches and are labeled by colors as indicated by the color band underneath the tree. (**B**) Heatmap of correlations followed by the *P*-values in parentheses between modules and development stage. The color of each square corresponds to the degree of correlation: positive correlation, red; negative correlation, green; no correlation, white. The “time” column on the left represents the correlation of each module with the entire developmental process.

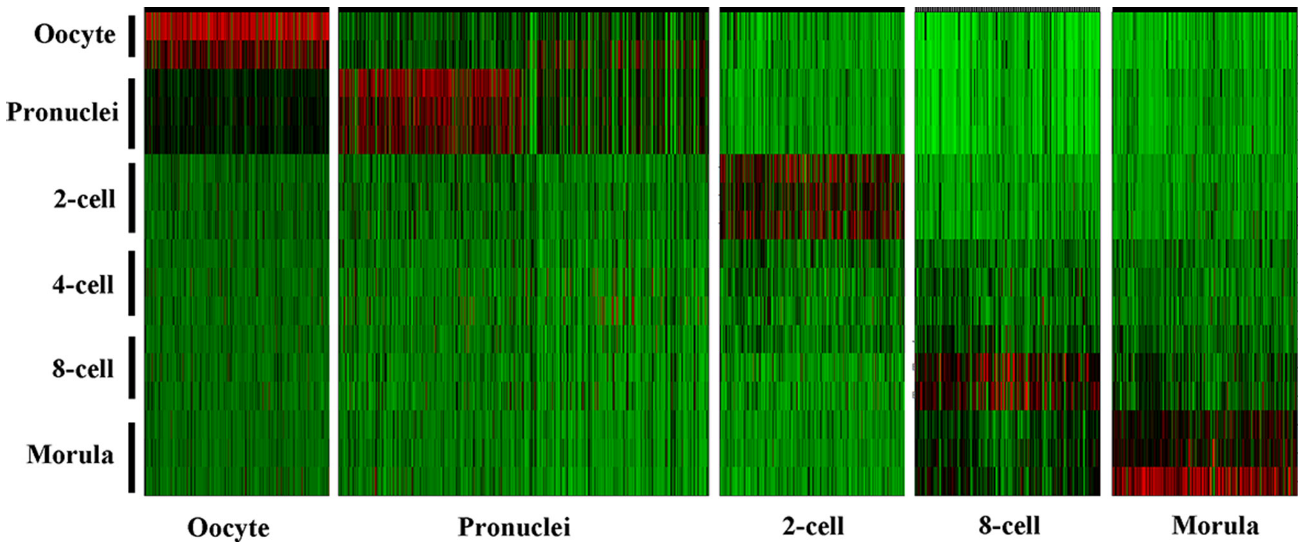

Supplementary Figure S6: Expression of mouse stage-specific genes. Heatmap showing expression pattern of mouse stage-specific genes in six representative stage-specific modules across all samples. The *x* axis shows the stage to which the module is highly correlated, and the *y* axis shows the whole developmental process.

Supplementary Table S1: Detail results of reads mapping and transcripts assembling. See Supplementary_Table_S1

Supplementary Dataset S1: The catalog of 421 novel lncRNAs. See Supplementary_Dataset_S1

Supplementary Dataset S2: Spearman correlation matrix derived from lncRNAs and coding genes in human dataset, respectively. See Supplementary_Dataset_S2

Supplementary Dataset S3: Spearman correlation matrix derived from lncRNAs and coding genes in mouse dataset, respectively. See Supplementary_Dataset_S3

Supplementary Dataset S4: The list of JS scores of lncRNAs and coding genes in human dataset. See Supplementary_Dataset_S4

Supplementary Dataset S5: The list of JS scores of lncRNAs and coding genes in mouse dataset. See Supplementary_Dataset_S5

Supplementary Dataset S6: Expression matrix of 15400 human stage-specific genes. See Supplementary_Dataset_S6

Supplementary Dataset S7: Expression matrix of 6063 mouse stage-specific genes. See Supplementary_Dataset_S7

Supplementary Dataset S8: Genes and hub genes of each human stage-specific modules. See Supplementary_Dataset_S8

Supplementary Dataset S9: Genes and hub genes of each mouse stage-specific modules. See Supplementary_Dataset_S9

Supplementary Dataset S10: GO and KEGG analysis of human stage-specific modules. See Supplementary_Dataset_S10

Supplementary Dataset S11: GO and KEGG analysis of mouse stage-specific modules. See Supplementary_Dataset_S11

Supplementary Dataset S12: Interaction analysis of hub genes of human stage-specific module. See Supplementary_Dataset_S12

Supplementary Dataset S13: Interaction analysis of hub genes of mouse stage-specific modules. See Supplementary_Dataset_S13
